# Ischaemic pre‐conditioning improves workload, fatigue and recovery in team sport: A randomized, double‐blind, placebo‐controlled crossover trial

**DOI:** 10.1113/EP093585

**Published:** 2026-05-21

**Authors:** Lorcan Daly, Shane Farrell, Alexander Gamble

**Affiliations:** ^1^ Department of Sport and Health Sciences Technological University of the Shannon Athlone Ireland; ^2^ SHE Research Centre Technological University of the Shannon Athlone Ireland; ^3^ Sport and Human Performance Research Centre University of Limerick Limerick Ireland; ^4^ Department of Physical Education and Sport Sciences University of Limerick Limerick Ireland

**Keywords:** blood flow restriction, exercise performance, fatigue, GPS workload, occlusion, recovery, recovery kinetics

## Abstract

Ischaemic preconditioning (IPC) is reported to improve performance, fatigue and recovery, yet evidence in team‐sport settings remains limited. We examined the effects of repeated IPC on external workload, perceptual strain and recovery during ecologically valid Gaelic football training. In a randomized, double‐blind, placebo‐controlled crossover design, 13 male players completed two identical pitch sessions following IPC (3 × 5 min 220 mmHg occlusions; 48 h, 24 h and 30 min pre‐session) or placebo. Global positioning system‐quantified external load, and perceptual outcomes (fatigue, soreness, differential RPE [overall, cognitive/technical, breathing, leg muscle]) were obtained pre‐ and 0, 24 and 48 h post‐exercise. Countermovement and drop jumps were assessed pre‐, post‐ and 48 h post‐exercise. Participants were naïve to both conditions. IPC resulted in larger very‐high‐speed running distance versus placebo (210.6 ± 220.9 vs. 81.6 ± 65.7 m; *P *= 0.046; effect size [ES] = 0.62; Bayes factor [BF_10_] = 1.71). Bayesian evidence also modestly supported greater sprint distance, high‐speed running and moderate‐intensity accelerations (BF_10_ = 0.38–0.41). Despite increased external loads, IPC lowered perceptual strain, namely physical fatigue, muscle soreness and cognitive effort (ES ≈ 0.20–0.40; BF_10_ = 0.28–0.49 [*P *> 0.05]). IPC also improved recovery, evidenced by significantly better countermovement jump outcomes (BF_10_ 1.53–3.21; *P *= 0.017–0.042). Other non‐significant outcomes were directionally consistent. This study provides evidence that repeated IPC can improve (i) on‐field workload, (ii) acute perceptual and physical strain/fatigue, and (iii) post‐exercise recovery in team‐sport athletes. Nevertheless, similar to other recovery interventions (e.g., ice baths), a theoretical risk of blunting fatigue/inflammation, and their subsequent adaptive signalling potential, implicates that application may be best periodized for phases prioritizing acute workload and/or recovery over physiological adaptation (e.g., in‐season, before important matches).

## Abstract (highlights)

- **What is the central question of this study?**
  Does repeated ischaemic preconditioning (IPC) enhance external workload, reduce perceptual fatigue/strain and accelerate recovery during ecologically valid team‐sport training?
- **What is the main finding and its importance?**
  IPC enabled team sport athletes to achieve greater on‐field workloads whilst experiencing lower perceptual strain and faster recovery, without apparent adverse effects. These findings suggest that IPC may be a useful, periodized strategy for enhancing acute performance capacity and recovery following team‐sport activity.

## 1 INTRODUCTION

During team sport match‐play/training, players execute challenging technical skills and engage in regular physical contests, whilst intermittently transitioning between low‐intensity (e.g., walking and jogging) and demanding high‐intensity actions, such as rapid high‐force changes of direction/pace, sprints, jumps and contests for possession (Daly et al., 2025; Koudellis et al., 2025; Mohr et al., 2023). These demands provoke substantial neuromuscular fatigue via factors such as metabolically mediated fatigue (Mohr et al., 2023; Panduro et al., 2022), lowered neural drive (Deely et al., 2022), as well as high cardiopulmonary and thermal strain (Henderson et al., 2023). Further, the high volumes of intense mechanical actions undertaken, many of which incorporate substantial eccentric components at high forces (Daly et al., 2025; Mohr et al., 2023), result in notable muscle damage (Deely et al., 2022). The conditions of match‐play/training acutely manifest progressive workload reductions (Duggan et al., 2024) and elicit significant psychophysiological disturbances, requiring longer periods for full recovery (Deely et al., 2022; Mohr et al., 2023). Therefore, the means with which to improve performance and/or recovery is highly valued (Daly et al., 2023b, 2024; Field et al., 2021; Mohr et al., 2022).

Ischaemic preconditioning (IPC), which involves brief cycles of perfusion and reperfusion, is widely reported to enhance athletic performance and/or limit fatigue (Caru et al., 2019; Incognito et al., 2016; Marocolo et al., 2025), as well as accelerate recovery (Daab et al., 2025; Patterson et al., 2021). Notably, reviews document that IPC elicits significant benefits for efforts principally constrained by oxidative capacity, such as time trial performances (e.g., 1–5 km), ${\bar{V}}_{O_{2}\mathrm{peak}}$, peak incremental power and time to exhaustion (Caru et al., 2019; Incognito et al., 2016; Marocolo et al., 2025). In these contexts, ∼3% improvements are observed on average, although responses are highly heterogeneous (Caru et al., 2019; Incognito et al., 2016; Marocolo et al., 2025), with various studies observing negligible or detrimental effects (Incognito et al., 2016). Key recent work from Mota et al. (2026), however, highlighted limited benefits following a bout of active or passive IPC in the Yo‐Yo Intermittent Recovery Test. However, notable inter‐individual variability and the limited evidence across ecologically valid training contexts, repeated IPC exposure and adult team sport populations highlight the need for further research.

Marocolo et al. (2025) outline possible determinant mechanisms explaining positive reported effects, such as improved mitochondrial respiration, blood flow (e.g., preservation of brachial flow‐mediated dilation and/or vascular conductance), hypoxia tolerance and red blood cell deformability, thereafter improving microcirculation. Beneficial effects of IPC on more intensive efforts are also documented, with reduced rate of torque loss (Pethick et al., 2020) and potential improvements in neural drive and motor cortex activation (Marocolo et al., 2025). Although data pertaining to neuromuscular performance are limited, available applied evidence suggests that IPC can improve jump height and reactive strength index outcomes (+3.6% to +7.8%) in track athletes (Gkari et al., 2025). These potential concurrent enhancements in aerobic capacity and neuromuscular performance are particularly relevant to team sport, since it is their combined role that determines physical performance (Daly et al., 2023a; Villaseca‐Vicuña et al., 2021). Also noteworthy is that IPC is demonstrated to lower fatigue (Daab et al., 2025) and accelerate post‐exercise recovery (Marocolo et al., 2025; Patterson et al., 2021). Therefore, recognising the considerable physical demands associated with contemporary training/competition schedules (Daly et al., 2023b, 2024; Harper et al., 2021) and prevalent implementation of recovery modalities in practice (Crowther et al., 2017; Field et al., 2021), the multifaceted effects of IPC merit investigation in team sport settings.

Whilst emerging evidence suggests that IPC may potentially augment recovery following various exercise stressors (Marocolo et al., 2025; Patterson et al., 2021), a comparatively larger body of work has investigated blood flow restriction applied post‐exercise (rather than as IPC *pre*‐exercise) with similar improved outcomes recorded (Oliva‐Lozano et al., 2024). For example, Patterson et al. (2021) applied IPC (3 × 5 min at 220 mmHg), either acutely or repeatedly over 3 days prior to a muscle damaging protocol of 5 × 20 60‐cm drop jumps (DJ), with an additional sham control group (Patterson et al., 2021). Principal among the findings were improved strength recovery, lowered muscle swelling, creatine kinase and muscle soreness in the IPC groups, with the largest benefits in the repeated IPC group (Patterson et al., 2021). Potentially explaining these findings, Marocolo et al. (2025) implicate candidate mechanisms such as lowered inflammatory cascades (via decreased redox signalling and upregulated antioxidant defences), improved myofibrillar organisation and upregulated membrane ion channel activity (Marocolo et al., 2025).

In contrast, other studies did not observe improved recovery outcomes with IPC, perhaps reflecting the high protocol variability in the literature base (Marocolo et al., 2025). Importantly, work applying pre‐exercise IPC in team sport training and match‐play contexts has been examined even less, with single studies demonstrating, respectively, (i) lowered fatigue immediately after a soccer simulation (Daab et al., 2025) and (ii) no difference in first half match workload in youth players (Vidigal et al., 2023). To the authors’ knowledge, research profiling post‐exercise recovery following direct team‐sport activity remains limited. Two studies in rugby union players provide useful insight, however, reporting (i) minimal benefits of IPC on acute recovery (1 h) after a simulated rugby protocol (e.g., jumps, passing, scrummaging, sprinting) in a small sample (*n *= 8, thereby plausibly limiting detection of small/moderate effects) or (ii) *post*‐exercise occlusion applied following sprints (5 × 50 m) in a larger cohort (*n *= 24) (Garcia et al., 2017; Williams et al., 2018). However, methodological variability/limitations, including sample size, variability in protocols/outcome measures, absence of true competitive small‐sided games or match‐play, use of sham conditions without blinding verification, and lack of repeated day IPC application, may restrict interpretation of these findings.

Notwithstanding the lack of competitive team sport‐relevant IPC research, it is critical to acknowledge major limitations of the broader IPC performance/recovery literature. These include prevalent (i) parallel study designs, which fail to account for individual differences like recovery kinetic responses or physical conditioning attributes (Daly et al., 2022; Oliva‐Lozano et al., 2024; Patterson et al., 2021), and (ii) absence of any adequate placebo control (Marocolo et al., 2015; Sabino‐Carvalho et al., 2017). Regarding the latter, the vast majority of studies apply a sham condition, most commonly involving cuffs placed and inflated to ≤20 mmHg (Incognito et al., 2016; Marocolo et al., 2025). This plausibly delivers questionable blinding efficacy (Marocolo et al., 2015). Further, studies have not assessed or reported participants’ perceptions of the blinding protocol, rendering placebo control adequacy impossible. This is problematic given that placebo effects have been evidenced to yield small‐to‐moderate effect size (ES) performance improvements (Hurst et al., 2020; Sabino‐Carvalho et al., 2017), with some showing ∼6% (ES = 1.2) enhancements (Clark et al., 2000). These magnitudes match or exceed those attributed to IPC interventions, raising concerns that observed IPC effects could potentially be largely placebo‐driven (Brown et al., 2023). In line with earlier suggestions (Brown et al., 2023), such methodological shortcomings significantly undermine confidence in the efficacy of IPC.

Accordingly, the present study aimed to assess the effects of IPC on physical performance, fatigue and recovery in team sport athletes under ecologically valid training conditions, whereby a standardised, in‐season session was designed and delivered by the teams coaching staff themselves and repeated identically across a 1‐week crossover period. For this study a randomized, double‐blind, placebo‐controlled crossover design was applied. Whilst the data regarding the effects of IPC are novel to any team sport, this study also provides applicable profiling of training load and responses in Gaelic football, delivering valuable insights for applied practice in this and other team sports with comparable demands

## 2 METHODS

### 2.1 Ethical approval

Ethical approval for this study was granted by the Faculty of Education and Health Sciences Research Ethics Committee at the University of Limerick (reference number: 2023_06_27_EHS). All procedures were conducted in accordance with the *Declaration of Helsinki*, except for registration in a database. Written informed consent was obtained from all participants prior to participation.

### 2.2 Experimental overview

This randomized, double‐blind, placebo‐controlled crossover trial investigated the effects of ischaemic preconditioning (IPC) on performance, fatigue and recovery in male Gaelic football players. Sixteen players were enrolled, with 13 completing the full protocol (one excluded due to injury, two due to missing assessment points). A familiarisation session preceded interventional data collection and included baseline familiarisation for warm up, countermovement jump (CMJ), DJ and perceptual assessments. Following this familiarisation and pre‐intervention data collection, participants were randomly allocated to begin with either the IPC or placebo condition using a counterbalanced method, with a 1‐week washout between conditions. Players were informed that they would receive two different sports performance interventions, each of which could potentially improve workload and/or recovery. The placebo was presented as a sports supplement, rather than a sham IPC, to enhance the credibility of the condition and avoid the blinding limitations associated with low‐pressure cuff inflation. None of the participants had prior knowledge of IPC or recognised it as a recovery or performance‐enhancing method (all indicating no preconceived expectations about whether, or to what extent, either intervention would influence performance or recovery).

To ensure an ecologically valid in‐season, competitive training session representative of normal field‐based team sport activity, and to maintain comparability between conditions, the coaching staff were instructed to design and deliver two identical training sessions during the crossover period. Both sessions were designed to replicate typical in‐season Gaelic football preparation, incorporating the same technical drills, small‐sided games, high‐intensity conditioning elements and work‐to‐rest ratios. Coaches were asked to identically match all activities, drill sequencing, intensities and durations as tightly as possible across conditions to minimise variability in external load and ensure that any observed differences could be attributed to the intervention rather than inconsistencies in training content. The complete session plan is provided in the current study's open access link along with its underpinning data (https://tinyurl.com/3hncnn9a) and the resultant external workload outputs are summarised in Table 3. Both training sessions were conducted on the same pitch and at the same time of day and day of the week (within the same microcycle format) to further standardise environmental and contextual factors. Nevertheless, any remaining random differences in session characteristics, which could occur even with identical planning as was presently applied, are inherently controlled for by the randomised, counterbalanced crossover design ensuring that each participant acted as their own control. This design also helps account for known inter‐individual variability in recovery kinetics, biological responses and other potential between‐individual fluctuations in performance and fatigue (Daly et al., 2022), aligning with recent calls in sport science to apply counterbalanced/crossover trials to improve validity and increase statistical power/efficiency (Kowalski et al., 2025).

In the placebo condition, participants ingested two oral capsules per day containing inert cornflour for the three time points preceding each training session. This was presented as a commercially available sports supplement, rather than a sham IPC, to enhance credibility and avoid the blinding limitations associated with low‐pressure cuff inflation. Players were informed that the study aimed to compare two interventions, each of which could potentially improve workload and/or recovery, without being told which was hypothesised to be more effective.

In the IPC condition, participants completed three cycles of 5‐min bilateral occlusion at 220 mmHg, interspersed with 5‐min reperfusion periods, applied to both lower limbs. Bilateral occlusion cuffs (Occlusion Cuff Pro, Occlusion Cuff Ltd, Belfast, UK) were positioned proximally on both thighs to target the lower‐limb musculature (e.g., quadriceps, hamstrings, gastrocnemius and soleus). The protocol was administered 48 h, 24 h and 30 min prior to the training session. All IPC sessions were performed with participants seated. The two pre‐session applications (48 and 24 h) were conducted at home under remote supervision via video call with Investigator A, who verified correct cuff placement and inflation pressure.

Home‐based application at 48 and 24 h pre‐session was implemented to minimise participant burden (travel, time away from home, etc.) and assess the feasibility of remotely supervised IPC. Compliance with IPC (and placebo ingestion) was confirmed via self‐reported logs. The final IPC application (30 min pre‐training) was conducted in the dressing room under direct supervision. Participants were familiarised with cuff application procedures during a dedicated familiarisation session. Inflation to the target pressure (220 mmHg) was used as the operational indicator of occlusion, with procedures standardised across all participants to ensure consistency. Individualised occlusion pressure or direct verification of arterial occlusion (e.g., via Doppler ultrasound) was not assessed, representing a methodological limitation. However, the protocol was specifically selected to replicate the repeated 3 × 5 min occlusion protocol at 220 mmHg described by Patterson et al. (2021), wherein this repeated daily IPC application demonstrated significant recovery benefits, and those effects were larger than a single pre‐session IPC exposure.

**FIGURE 1:**
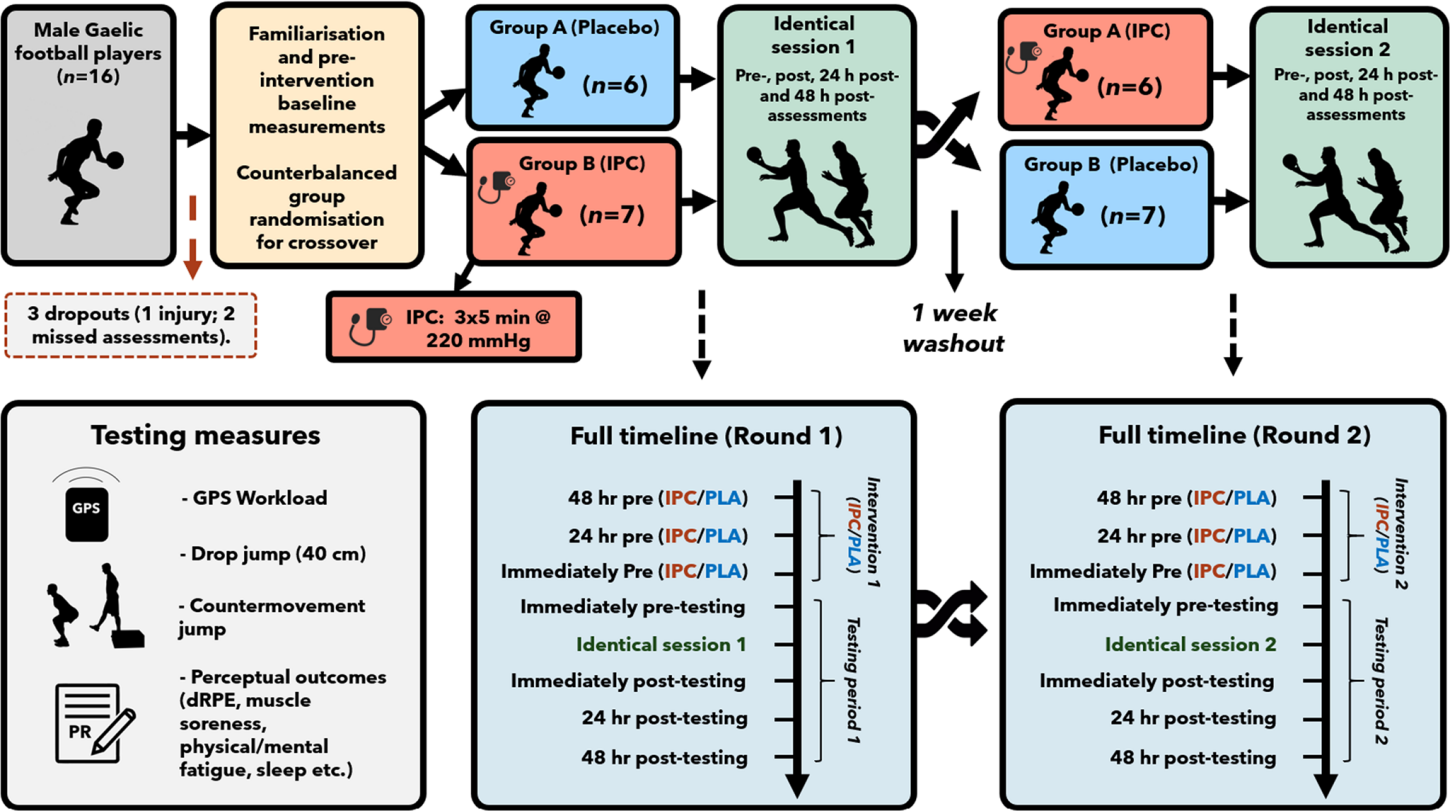
Schematic overview of study design.

CMJ and DJ were assessed pre‐training, immediately post‐training and 48 h post‐training. Perceptual outcomes, covering physical and mental fatigue, soreness, sleep, mood and rate of perceived exertion (RPE) were assessed at all four time points (30 min pre‐, immediately post‐, 24 h and 48 h post‐training). Global positioning system (GPS) data were recorded during training to quantify external workload. Investigators B and C, who conducted all physical testing, were blinded to group allocation, while only Investigator A managed the intervention assignments, ensuring maintenance of researcher blinding and minimisation of potential tester bias (subconscious cuing, etc.). Figure 1 presents a schematic overview of the study.

### 2.3 Participants

Thirteen active male Gaelic football players (age: 21.9 ± 3.2 years; height: 176.6 ± 5.9 cm; body mass: 80.5 ± 14.3 kg) were recruited from a single senior club‐level Gaelic football club. All participants had a minimum of 2 years’ playing experience and were training regularly at least 2–3 days week^−1^. Exclusion criteria included any lower‐body injury or major injury sustained in the previous 2 months. None of the participants had prior knowledge of IPC or were informed of the specific rationale of the study. A familiarisation session was conducted 1 week prior to data collection, which included baseline CMJ testing and completion of questionnaires. Participants completed a PAR‐Q before commencing the familiarisation session, and were informed that IPC may cause mild discomfort, though blood flow restriction is widely considered a safe intervention with minimal side effects (Patterson et al., 2019). Researchers monitored participants throughout the IPC protocol, in person (on site) or via an online video meeting (when undertaken remotely) to ensure safety, comfort and correct application.

### 2.4 Countermovement and drop jump

A standardized warm‐up preceded all testing points and included light jogging (2 min) followed by dynamic movements relevant to CMJ execution (e.g., lunges, squats, hip thrusts, sub maximal hops), in line with recommendations (Bishop, 2003). During all testing procedures, standardised instructions were delivered to all participants, eliminating the known cofounder of instruction variability (Khuu et al., 2015). Participants completed three familiarisation jumps before resting for 2 min, then completing CMJ, DJ and perceptual tests. CMJs were performed on dual Force Plates (ForceDecks, VALD Performance, Newstead, Australia). Similar to earlier work (Hughes et al., 2022), raw vertical ground reaction force data were collected and analysed using ForceDecks software, which automatically identifies key temporal events (i.e., movement onset, braking phase, propulsion phase and take‐off), subsequently computing standard kinetic/kinematic CMJ variables. Participants were instructed to place hands on hips, assume an upright stance, and perform a rapid CMJ with full lower‐limb extension via self‐selected depth, aiming to ‘jump as high as possible’ before landing in a stable manner, similar to earlier methods (Daly et al., 2023a). A rest period of 45 s was given between trials, whereby three trials were recorded and their subsequent average was used for analysis (Daly et al., 2022). The following CMJ variables were extracted from the force–time data as per VALD software computations. Specifically, jump height (JH) was calculated using the impulse–momentum method, wherein net impulse above body weight (force × time) was integrated to determine vertical take‐off velocity, from which JH was derived via the kinematic equation JH = *v* ^2^/(2×gravity [9.81]), where *v* is the calculated vertical take‐off velocity. Peak power was defined as the highest instantaneous power output (force × velocity) achieved during the concentric phase. As described previously by Hughes et al. (2022), readers should consider that power is a scalar quantity and therefore has magnitude but no direction. Because the values reported in this study pertain to vertically oriented mechanical output during the CMJ, they represent directional projections rather than true scalar power. Accordingly, these values are best interpreted as *quasi‐power* measures, reflecting vertical axis braking/propulsion power (Hughes et al., 2022). Concentric mean force and eccentric mean force represented the average vertical force produced during the propulsion and braking phases, respectively. Concentric mean power and eccentric mean power quantified mean power output across the concentric and eccentric phases. Reactive strength index–modified (RSI_mod_) was computed as JH divided by time to take‐off. Contraction time was defined as the total duration from movement onset to take‐off. Variables were exported directly from VALD ForceDecks for subsequent analysis. The DJ and CMJ were performed and recorded using a photoelectric optical measurement device and accompanying software (Optojump, Microgate, Bolzano, Italy).

DJ testing was completed using the same protocol as methods previously described (Daly et al., 2022). Herein, participants stood on a 40 cm‐high step with their hands placed on their hips before stepping off using their preferred foot first, with both feet simultaneously landing on the ground, before immediately jumping vertically as high as possible. In line with recommendations (Khuu et al., 2015), participants were instructed to (i) not lift their centre of gravity during the step‐off phase and (ii) jump to a maximal height while minimizing ground contact time.

### 2.5 Perceptual responses

Participants completed self‐reported perceptual measures at four time points: pre‐training, immediately post‐training, 24 h post‐training and 48 h post‐training. At each time point, participants completed a battery of self‐reported measures on a sheet of paper. This included (i) a 5‐item Likert scale questionnaire as per McLean et al. (2010) assessing overall recovery (sleep quality, fatigue, muscular soreness, stress level and mood) and (ii) visual analogue scales (0–100) assessing (i) mental and physical as per Russell et al. (2021), muscle soreness as per Patterson et al. (2021) and differential RPE (dRPE) assessing overall, leg muscle, breathlessness and technical/tactical RPE as per McLaren et al. (2017). All perceptual scales were completed individually and participants were instructed to stand at least 2 m away from support staff and players during on‐site administration to minimise peer or social influence on responses (McLaren et al., 2017; McLean et al., 2010). Participants were familiarised with each scale and provided with standardised explanations to ensure clarity and consistent interpretation across data collection points.

### 2.6 Statistical analysis

Descriptive statistics (means ± SD) were calculated for all anthropometric, GPS workload, perceptual and neuromuscular variables. Given the randomized, double‐blind, placebo‐controlled crossover design, the primary comparison of interest was the effect of condition (IPC vs. placebo) across repeated time points. Participants were allocated in a counterbalanced order using the Excel randomisation function (RAND), ensuring an equal distribution of starting conditions. To examine condition effects on fatigue and recovery responses, analysis of covariance (ANCOVA) with Bonferroni‐adjusted *post hoc* tests were used, with baseline pre‐session values entered as covariates. This approach controlled for any pre‐intervention differences in CMJ, DJ, perceptual or GPS measures. Within‐condition changes across time (pre, post, 24 h, 48 h) were assessed using repeated‐measures ANOVA with Bonferroni *post hoc* tests, and Cohen's effect size (ES) used to characterise the magnitude of change. Effect sizes were interpreted as: trivial (<0.20), small (0.20–0.60), moderate (0.61–1.19), and large (>1.20). Statistical significance was accepted at α = 0.05. Additional Bayesian *t*‐tests and ANCOVAs were conducted to provide a continuous measure of the strength of evidence for or against IPC compared with placebo. Results were interpreted in line with the classification scheme reported by Lee & Wagenmakers (2014): (i) *for the alternative hypothesis* (H1) where Bayes factor (BF_10_) = 1–3 indicates anecdotal evidence; 3–10, moderate evidence; 10–30, strong evidence; 30–100, very strong evidence; and >100, extreme evidence; and (ii) *for the null hypothesis* (H0) where BF_10_ = 0.33–1 indicates anecdotal evidence; 0.10–0.33, moderate evidence; 0.03–0.10, strong evidence; 0.01–0.03, very strong evidence; and <0.01, extreme evidence. All analyses were conducted using JASP (Version 0.19.0, University of Amsterdam, Amsterdam, the Netherlands). All figures present raw datapoints, individual and group temporal trajectories, means ± SD and distribution clouds to provide a comprehensive view of individual and collective responses.

## 3 RESULTS

Participants rated both interventions similarly prior to experiencing them (no knowledge of either following explanation), consistent with successful blinding. Notably, following the interventions, players reported via a remote anonymous online questionnaire (to help eliminate responder bias) perceived benefits of both placebo and IPC conditions, with a greater perceived benefit for IPC compared with the placebo condition (1, totally ineffective; 10, extremely effective; IPC 7.0 ± 1.3 vs. placebo [PLA] 4.0 ± 2.4; *P* = 0.001), also reflected in higher ratings for likelihood of future use (1, definitly would not use; 10, definitely would use; IPC 7.4 ± 1.9 vs. PLA 4.5 ± 2.7; *P* = 0.006).

All analysed outcomes are reported in the accompanying tables and figures, with statistical significance for within‐ (temporal) and between‐condition differences indicated within the tables. The raw data are also available at the open‐access link provided (https://tinyurl.com/3hncnn9a) for verification and/or reuse. Table 1 presents perceptual outcomes across all time points for both conditions, Table 2 summarises GPS‐derived external load metrics from the training sessions and Table 3 reports all CMJ and DJ neuromuscular outcomes.

**TABLE 1:** External load data recorded by global positioning system (GPS) during the training sessions for both conditions.

Variable	PLA, mean ± SD	IPC, mean ± SD	ES	*P*	BF_10_
Distance (m)	5145.4 ± 526.1	5301.46 ± 574.35	0.180	0.529	0.334
Moderate speed or greater (>4 m s^−1^) distance (m)	1096.6 ± 912.8	1309.8 ± 627.7	0.272	0.494	0.352
High speed or greater (>5.5 m s^−1^) distance (m)	407.6 ± 317.5	556.5 ± 412.9	0.233	0.417	0.376
Very high speed (>6 m s^−1^) distance (m)	81.6 ± 65.7	**210.6 ± 220.9**	0.616	**0.046**†	1.714
Max speed (m s^−1^)	7.54 ± 0.39	7.6 ± 0.51	0.049	0.861	0.282
Sprint (>7.2 m s^−1^) distance (m)	30.4 ± 49.9	56.6 ± 70.1	0.298	0.304	0.452
Moderate (>2.5 m s^−2^) or greater intensity accels (*n*)	20.1 ± 11.8	23.9 ± 9.3	0.361	0.366	0.410
Moderate (>−2.5 m s^−2^) or greater intensity decels (*n*)	22.2 ± 7.9	22.4 ± 11.7	0.023	0.953	0.279
High intensity (>3.5 m s^−2^) accels (*n*)	4.2 ± 4.8	5.5 ± 3.9	0.172	0.547	0.329
High intensity (>−3.5 m s^−2^) decels (*n*)	6.2 ± 3.3	6.1 ± 5.3	0.017	0.965	0.279
Total session time (s)	4162.9 ± 159.0	4157.5 ± 129.7	−0.031	0.913	0.280

*Note*: Values presented as means ± SD. †Significant between‐condition differences indicated in bold, *P *< 0.05. BF_10_, Bayes factor; EF, effect size; IPC, ischaemic preconditioning; PLA, placeb.

**TABLE 2:** Perceptual responses across time for both conditions.

	PLA, mean ± SD	ES	IPC, mean ± SD	ES	*P*	BF_10_
Physical fatigue						
Pre	33.8 ± 22.5	—	44.0 ± 22.6	—	—	—
Post	61.2 ± 18.3*	−1.194	60.9 ± 20.0*	−0.769	0.503	0.407
24	45.7 ± 23.7	−0.518	39.8 ± 21.8	0.193	0.278	0.476
48	50.3 ± 26.7	−0.719	39.2 ± 23.2	0.221	0.166	0.626
Mental fatigue						
Pre	35.6 ± 20.1	—	43.0 ± 24.02	—	—	—
Post	45.2 ± 18.3	−0.443	50.1 ± 21.39	−0.324	0.921	0.376
24 h	43.5 ± 24.5	−0.361	37.8 ± 21.91	0.240	0.300	0.489
48 h	44.2 ± 23.4	−0.397	32.2 ± 19.82	0.493	0.078	0.925
						
Muscle soreness						
Pre	36.3 ± 23.7	—	34.9 ± 24.3	—	—	—
Post	61.2 ± 19.9*	−1.009	53.0 ± 21.7*	−0.786	0.280	0.562
24 h	48.2 ± 25.4	−0.483	40.8 ± 19.7	−0.254	0.414	0.472
48 h	47.3 ± 29.0	−0.445	41.5 ± 25.8	−0.288	0.612	0.401
Total recovery						
Pre	17 (15, 19)	—	16 (15, 19)	—	—	—
Post	15 (14, 16)	—	15 (15, 17)	—	—	0.978
24	16 (15, 19)	—	19 (15, 19)	—	—	0.372
48	19 (15, 20)	—	17 (14, 19)	—	—	0.374
RPE	62.6 ± 21.9	—	60.7 ± 22.3	0.091	0.748	0.292
Cognitive RPE	58.1 ± 19.6	—	48.39 ± 21.8	0.326	0.263	0.494
Leg muscle RPE	65.9 ± 25.5	—	57.85 ± 23.4	−0.077	0.844	0.465
Breathing RPE	61.2 ± 25.8	—	58.31 ± 28.2	0.209	0.529	0.303

*Note*: Values presented as means ± SD, except for total recovery score (non‐parametric), which is reported as median (interquartile range). *P*‐value from ANCOVA for physical fatigue, mental fatigue, muscle soreness and total recovery; *P*‐value by *t*‐test for RPE. Statistical significance for within‐condition (time) effects are indicated in bold, *P < 0.05. BF_10_, Bayes factor; EF, effect size; IPC, ischaemic preconditioning; PLA, placebo; RPE, rate of perceived exertion.

**TABLE 3:** Countermovement and drop jump outcomes across time for both conditions.

CMJ variable	PLA, mean ± SD	ES	IPC, mean ± SD	ES	ANCOVA *P*	BF_10_
Jump height (cm)						
Pre	32.1 ± 4.7	—	30.3 ± 4.3	—	—	—
Post	31.1 ± 4.5	0.221	30.5 ± 4.2	−0.056	0.311	0.523
48 h post	30.5 ± 5.0	0.337	31.7 ± 4.1	−0.341	**0.042†**	1.535
Positive impulse (Ns)						
Pre	545.7 ± 111.7	—	532.3 ± 111.6	—	—	—
Post	537.6 ± 114.9	0.072	534.6 ± 111.2	−0.021	0.285	0.550
48 h post	518.0 ± 107.4*	0.248	538.5 ± 109.2	−0.056	**0.017†**	3.207
Peak power (W)						
Pre	3968.3 ± 911.9	—	3734.4 ± 863.9	—	—	—
Post	3835.9 ± 862.8	0.152	3753.9 ± 808.8	−0.024	0.077	1.120
48 h post	3819.0 ± 830.3	0.172	3841.0 ± 816.0	−0.128	**0.030†**	2.008
Concentric force avg. (N)						
Pre	1514.5 ± 331.3	—	1466.1 ± 318.5	—	—	—
Post	1485.9 ± 323.2	0.089	1460.3 ± 320.5	0.019	0.515	0.443
48 h post	1466.9 ± 312.8	0.147	1471.7 ± 305.8	−0.018	0.086	1.080
Eccentric force avg. (N)						
Pre	790.5 ± 141.5	—	790.21 ± 140.79	—	—	—
Post	784.0 ± 140.9*	0.046	783.9 ± 140.8**	0.044	0.902	0.351
48 h post	790.5 ± 142.5	<0.001	789.3 ± 141.1	0.007	0.647	0.385
Concentric power avg.(W)						
Pre	2079.42 ± 555.76	—	1967.9 ± 539.2	—	—	—
Post	2015.39 ± 545.31	0.559	1958.4 ± 522.3	0.057	0.401	0.457
48 h post	1952.6 ± 504.8*	0.625	2011.5 ± 523.4	−0.371	**0.019†**	2.869
Eccentric power avg. (W)						
Pre	408.1 ± 132.5	−	417.1 ± 133.6	—	—	—
Post	417.3 ± 131.9	−0.072	417.1 ± 134.5	<0.001	0.706	0.381
48 h post	393.6 ± 113.5	0.115	433.5 ± 117.2	−0.128	0.098	1.006
RSI modified (m s^−1^)						
Pre	0.39 ± 0.1	—	0.34 ± 0.1	—	—	—
Post	0.38 ± 0.1	0.066	0.36 ± 0.1	−0.211	0.473	0.428
48 h post	0.35 ± 0.1	0.310	0.37 ± 0.1	−0.247	0.113	0.492
Contraction time (ms)						
Pre	881.4 ± 185.9	—	931.8 ± 166.5	—	—	—
Post	867.9 ± 156.6	0.082	886.8 ± 148.0	0.279	0.792	0.373
48 h post	921.3 ± 149.0	−0.324	910.8 ± 169.3	0.130	0.470	0.417
Drop jump variables						
Drop jump RSI						
Pre	1.16 ± 0.38	—	1.10 ± 0.32	—	—	—
Post	1.13 ± 0.33	0.098	1.16 ± 0.27	−0.198	0.212	0.629
48 h post	1.16 ± 0.28	0.005	1.19 ± 0.31	−0.295	0.405	0.455
Drop jump height (cm)						
Pre	27.4 ± 6.6	—	25.8 ± 6.0	—	—	—
Post	27.0 ± 5.7	0.084	26.9 ± 5.0	0.437	−0.200	0.551
48 h post	27.3 ± 4.8	0.023	27.9 ± 5.3	0.570	−0.382	0.567
Drop jump contact time (s)						
Pre	0.246 ± 0.056	—	0.241 ± 0.044	—	—	—
Post	0.246 ± 0.044	0.003	0.236 ± 0.034	0.110	0.473	0.444
48 h post	0.241 ± 0.032	0.120	0.246 ± 0.070	−0.092	0.625	0.395

*Note*: Values presented as means ± SD. Statistical significance for within‐condition (time) effects is indicated in bold, *P < 0.05, **P < 0.01. Between‐condition (ANCOVA) effects are indicated in bold, †*P *< 0.05. BF_10_, Bayes factor; CMJ, countermovement jump; ES, effect size; IPC, ischaemic preconditioning; PLA, placebo; RSI, reactive strength index.

### 3.1 External workload

IPC resulted in a significant increase in very high‐speed running distance (>6 m s^−^^1^) compared with placebo (210.6 ± 220.9 vs. 81.6 ± 65.7 m; +158.1%; *P* = 0.046; ES = 0.616; BF_10_ = 1.714). Although directionally consistent, no significant between‐condition differences were observed for the remaining external load variables, whereby IPC presented higher but non‐significant values in all outcomes except high‐intensity decelerations. For remaining non‐significant outcomes, effect sizes ranged from 0.017 to 0.361 in favour of IPC versus placebo for all outcomes (+0.8 to 86.2%), except high‐intensity decelerations (−1.6%), with BF_10_ values ranging from 0.279 to 0.452 across variables.

### 3.2 Immediate post‐session acute responses

No significant between‐condition differences were observed for any perceptual fatigue or RPE‐derived outcomes, although effect sizes ranged from −0.786 to −0.324 across perceptual outcome, indicating more favourable responses for IPC versus placebo. BF_10_ values ranged from 0.292 to 0.562 across variables. Regarding perceptual responses (soreness, total recovery, physical/mental fatigue), these were consistently more favourable with IPC (+16.5% to +51.9%) versus placebo (+27.0% to +81.1%), indicating an attenuated acute fatigue response with IPC. Similarly, total and differential (leg muscle, breathlessness, cognitive) RPE were lower in the IPC condition (−3 to −16.7%).

Regarding CMJ and DJ outcomes immediately post‐exercise, there were no statistically significant between‐condition differences. Pre‐ to post‐exercise effect sizes ranged from −0.211 to 0.559, with BF_10_ values ranging from 0.351 to 0.706 across variables. Notably, most jump outcomes (9/12; 1/12 identical) demonstrated more favourable post‐exercise changes following IPC (−0.5% to +5.9%), whereas fewer variables favoured placebo (2/12; −1.5% to +2.3%).

### 3.3 Recovery responses 24 and 48 h post‐session

IPC resulted in significant between‐condition effects at 48 h post‐exercise for several CMJ‐derived outcomes in favour of IPC, including jump height (+4.6%; *P *= 0.042; ES = −0.341; BF_10_ = 1.535), positive impulse (+1.2%; *P *= 0.017; ES = −0.056; BF_10_ = 3.207), peak power (+2.8%; *P *= 0.030; ES = −0.128; BF_10_ = 2.008) and concentric mean power (+2.2%; *P *= 0.019; ES = −0.371; BF_10_ = 2.869).

Between‐group effects were non‐significant from pre‐ to 24 and 48 h for all perceptual, DJ and remaining CMJ outcomes. Herein, from pre‐ to 24 and 48 h post‐exercise, perceptual physical fatigue, mental fatigue and muscle soreness outcomes increased to a greater extent in placebo (+22.2% to +48.8%), whereas IPC demonstrated attenuated fatigue/soreness responses (−25.1% to +18.9%), including improvements in physical and mental fatigue versus pre‐exercise. From pre‐ to 48 h post‐exercise, 8/9 CMJ‐derived outcomes demonstrated superior recovery in IPC versus placebo, with percentage changes ranging from −0.1% to +8.8% in IPC and −10.3% to 0.0% in placebo, with one variable showing identical responses between conditions. Similarly, 2/3 drop jump outcomes favoured IPC, with improvements of approximately +8.1% to +8.2%, whereas drop jump contact time favoured placebo (−2.0% vs. +2.1%). Effect sizes ranged from −0.378 to 0.493 principally in favour of IPC, with BF_10_ values ranging from 0.372 to 1.080 across variables.

**FIGURE 2:**
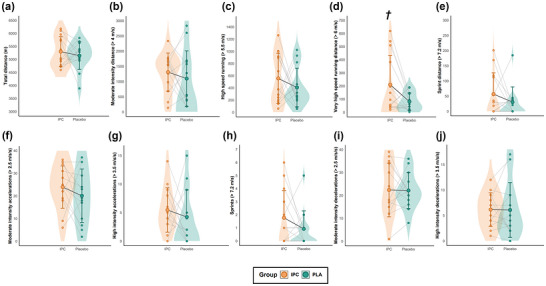
IPC versus placebo workload outcomes for (a) total distance, (b) moderate‐intensity distance (>4 m s^−^^1^), (c) high‐speed running distance (>5.5 m s^−^^1^), (d) very high‐speed running distance (>6 m s^−^^1^), (e) sprint distance (>7.2 m s^−^^1^), (f) moderate‐intensity accelerations (>2.5 m s^−^^2^), (g) high‐intensity accelerations (>3.5 m s^−^^2^), (h) sprints (>7.2 m s^−^^1^), (i) moderate‐intensity decelerations (>2.5 m s^−^^2^), and (j) high‐intensity decelerations (>3.5 m s^−^^2^). †Statistical significance for between‐condition difference in workload outcome, *P *< 0.05. All outcomes: *n *= 13.

**FIGURE 3:**
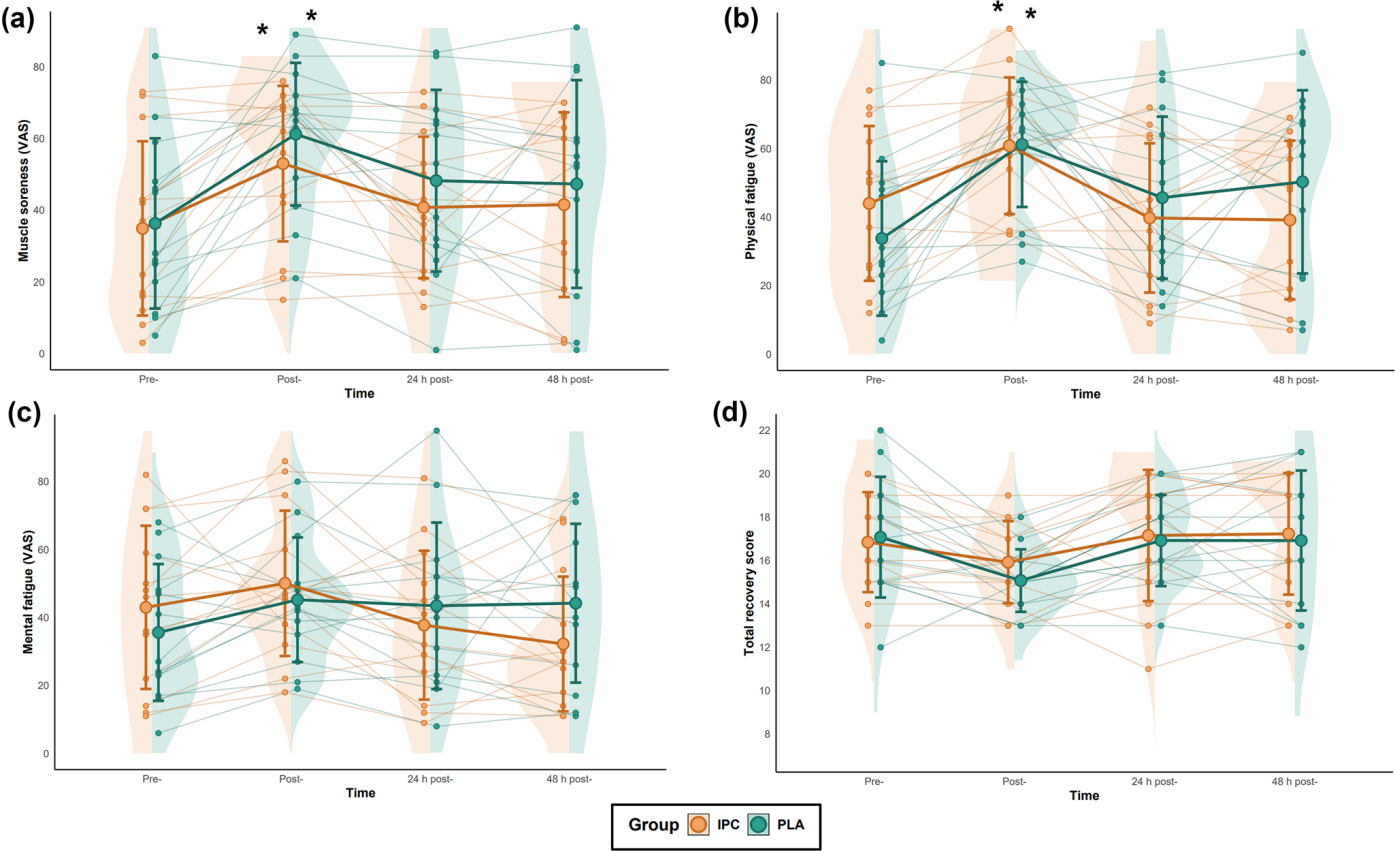
IPC versus placebo perceptual outcomes for (a) muscle soreness, (b) physical fatigue, (c) mental fatigue and (d) total recovery score (reverse scoring [higher score, less recovery]). Statistical significance for within‐condition effect over time is indicated, *P < 0.05, **P < 0.01. All outcomes: *n *= 13. VAS, visual analogue scale.

**FIGURE 4:**
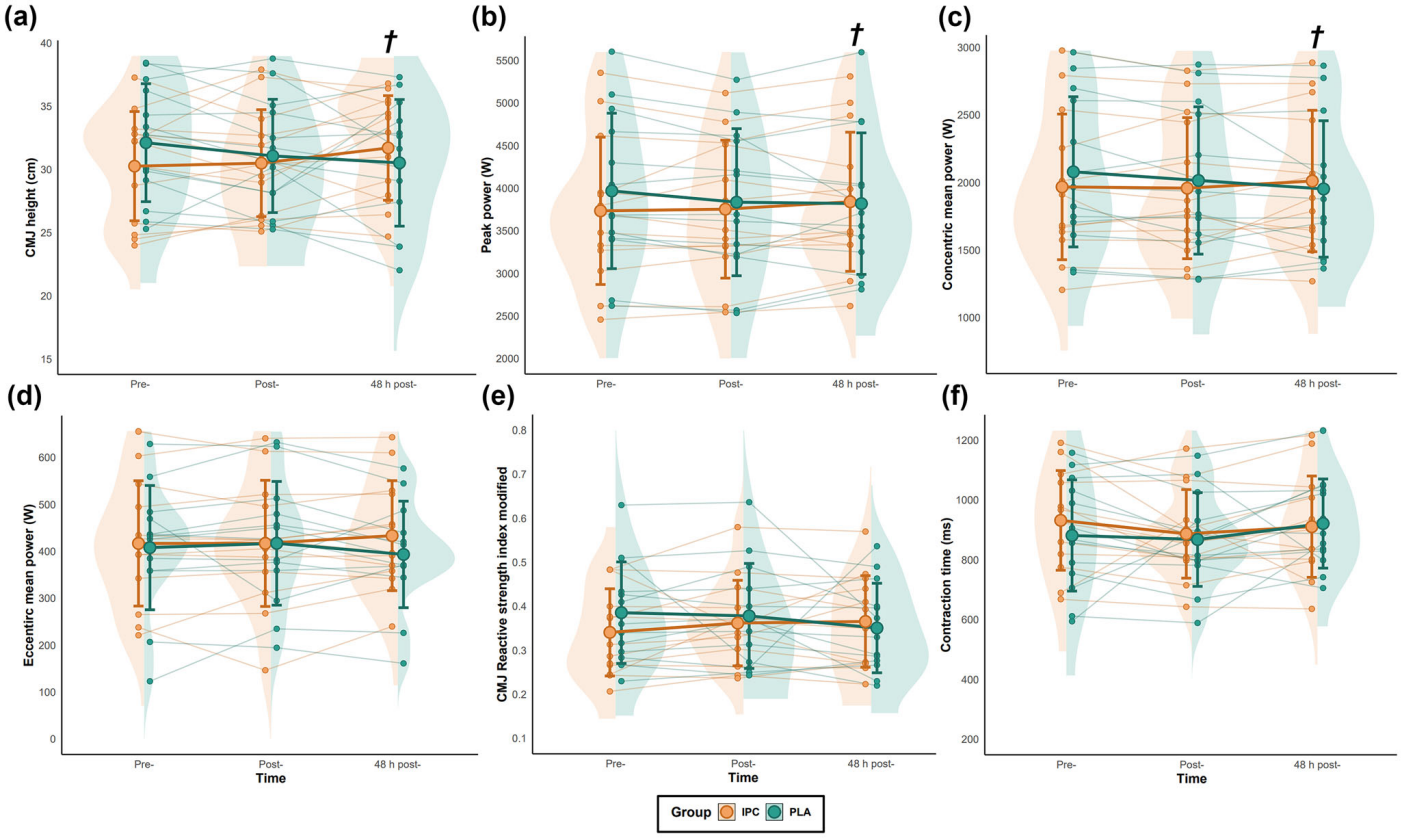
IPC versus placebo countermovement jump (CMJ) outcomes for CMJ (a) height, (b) peak power, (c) concentric ‘upward’ mean power, (d) eccentric ‘downward’ mean power, (e) reactive strength index modified, and (f) contraction time. †Between‐condition (ANCOVA) effects, *P *< 0.05. All outcomes: *n *= 13.

**FIGURE 5:**
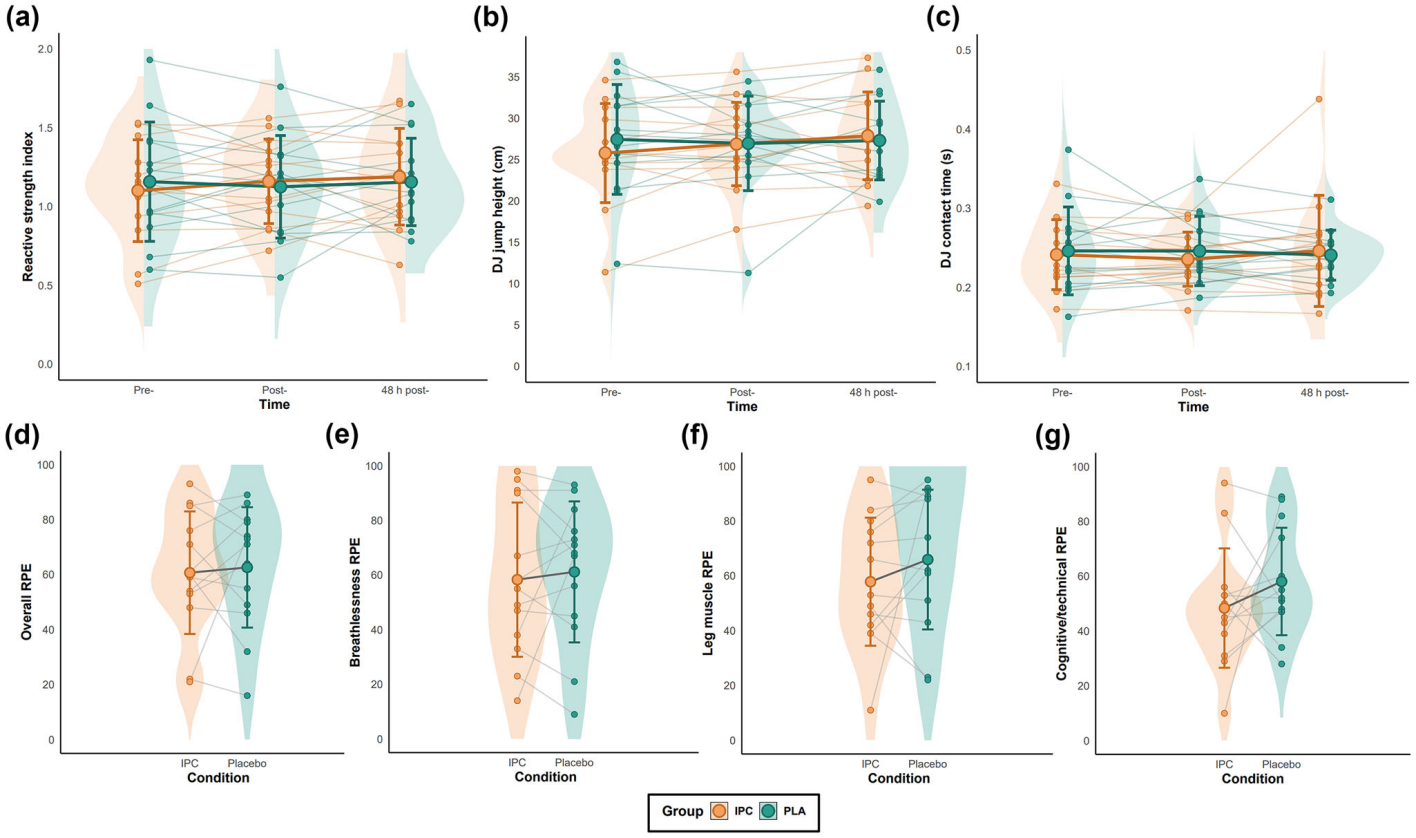
(a–c) IPC versus placebo for drop jump: (a) reactive strength index, (b) height, and (c) contact time. (d–g) IPC versus placebo for RPE: (d) overall, (e) breathlessness, (f) leg muscle, and (g) cognitive/technical. All outcomes: *n *= 13.

Figures 2, 3, 4, 5 display the raw datapoints for each variable, distribution clouds, individual athlete trajectories and group mean ± SD lines, providing a visual representation of both individual responses and overall trends.

## 4 DISCUSSION

This study investigated the effects of IPC on physical performance, fatigue and recovery in Gaelic football players under ecologically valid training conditions, using a randomized, double‐blind, placebo‐controlled crossover design. The primary findings were that there was (i) enhanced external workload (e.g., total distance, high‐speed running and accelerations), (ii) similar or lower perceptual disturbances, and (iii) improved post‐training recovery markers, including jump performance outcomes, perceived fatigue and muscle soreness, despite undertaking more strenuous workloads in the IPC group compared to the placebo group. This is one of the first studies to suggest that repeated IPC may offer multifaceted benefits related to performance and post‐exercise fatigue and muscle damage.

Foremost, IPC resulted in elevated on‐field workload, with significantly greater very high‐intensity running distances (*P *= 0.046; ES = 0.616) observed compared to placebo. For instance, whilst total (3.0 ± 0.9%) and high‐speed (36.5 ± 23.5%) showed small‐to‐moderate effect sizes in favour of IPC versus placebo, statistical significance was not reached. Nonetheless, the observed increase in very high‐intensity running aligns with previous research indicating that IPC enhances work capacity, which has been suggested as factors such as oxygen delivery and utilization efficiency during exercise (Caru et al., 2019) or pain tolerance (Marocolo et al., 2025). The apparent selective enhancement of high‐intensity workload indicated by these data is consistent with the ergogenic profile of IPC described in the literature base, spanning laboratory and field contexts (Caru et al., 2019; Daab et al., 2025; Marocolo et al., 2025). More work is needed to support these observed findings and potential mechanisms of action, but these preliminary insights agree with previously documented improvements in performance related to aerobic energy metabolism and neuromuscular performance outcomes, which may translate to increased work rates among team sport athletes. In contrast, the present findings differ from recent work in youth soccer players (Mota et al., 2026), where no improvements in YYIRT performance were observed following a bout of active or passive IPC (Mota et al., 2026). These differences may reflect variations in protocol (e.g., timing, repeated exposures, different exercise tasks), population, or contextual factors. Notably, considerable inter‐individual variability was also evident in the data from Mota et al. (2026), consistent with the present data, reinforcing the need for larger samples to improve the robustness of effect estimates and reduce outlier sensitivity.

Remarkably, despite the elevated high‐intensity workloads, IPC simultaneously resulted in acute reductions in RPE, cognitive load and leg discomfort immediately post‐session. This notable decoupling between external workload and internal strain provides evidence that IPC can confer benefits encompassing work capacity and fatigue resistance concurrently. In contrast, previous work in rugby union players has reported minimal effects of IPC on (i) acute (1 h) outcomes when applied prior to simulated activity (passing, scrummaging, a sprint, etc.) (Garcia et al., 2017) or (ii) applied *post*‐exercise following repeated sprint exercise (Williams et al., 2018), suggesting that such dissociations may be context‐dependent. It is possible that the small sample in the former study (*n *= 8) limited the ability to detect effects (Garcia et al., 2017), whilst the latter larger cohort (*n *= 24) still demonstrated limited benefits of post‐exercise occlusion applied after 5 × 50 m sprints. Additionally, potentially suboptimal placebo control (e.g., lack of blinding verification and use of minimal cuff inflation) may have influenced outcomes. Despite already presenting limited benefits, in such cases, a true placebo response would likely act to improve the control condition, thereby reducing the apparent effectiveness of IPC/occlusion further, contrasting with the present findings. Importantly, notable contextual differences (lack of competitive game‐play, single rather than repeated day IPC application, etc.) should also be considered and may help explain differences. In light of the present study's findings of modestly increased workload alongside reduced perceptual fatigue, the ability to sustain higher mechanical outputs at a similar/lower perceived effort may reflect IPC‐mediated influences on cardiovascular function, pain perception and/or central fatigue regulation (Incognito et al., 2016; Marocolo et al., 2025). Regarding the latter, possible contributors may include improved cortical excitability, central motor command efficiency, and reduced afferent feedback from type III/IV muscle afferents due to better microcirculatory function (Marocolo et al., 2025). Additionally, factors such as the early maintenance of muscle pH and delayed metabolite accumulation reported following IPC application, as outlined by Caru et al. (2019), may reduce the sensory drive contributing to perceived exertion, thereby allowing greater external work for the same or lower subjective effort, as documented herein (Figure 5). These effects would be of great interest for practitioners given the strenuous psychophysiological demands of intermittent team sport and application of performance‐ and recovery‐enhancing modalities (Daly et al., 2023b, 2024; Field et al., 2021).

Another notable finding was the favourable recovery profile seemingly elicited via IPC, which included neuromuscular (DJ and CMJ) and perceptual based factors. The effects noted could be conceivably larger if the IPC group undertook the same, rather than larger, workload as the placebo group. Improved recovery was evidenced by reduced physical fatigue and muscle soreness at 24–48 h, as well as superior CMJ height, peak power and eccentric mean power recovery at 48 h. These neuromuscular benefits are congruent with data from Patterson et al. (2021). Here, a faster restoration of maximal voluntary contraction, reduced creatine kinase release and lower muscle soreness was reported following repeated IPC when compared with sham control. Likewise, Daab et al. (2025) also observed reduced acute fatigue with IPC following soccer simulation, and the current data support these findings and the applied efficacy of IPC. However, other studies did not report significant recovery benefits (Cerqueira et al., 2021). Notably, heterogeneity in the literature regarding the magnitude of IPC's effects is likely reflective of differences in participant training status, exercise modality, dosing frequency, nocebo/placebo effects and/or possible individual response variability. Potentially explaining prior positive findings, IPC may limit inflammation and oxidative stress, helping preserve muscle structure, while also improving membrane and ion‐channel function to facilitate faster excitation–contraction coupling recovery (Marocolo et al., 2025; Patterson et al., 2021).

This study presents a number of novel findings, many of which should be of interest to and/or guide future research‐ and practice‐based endeavours. The combination of (i) increased high‐intensity workload, (ii) stable or reduced acute perceptions of fatigue, soreness or exertion, and (iii) accelerated recovery responses adds a compelling case for IPC as potentially useful tool in team sport settings under appropriate conditions. For instance, achieving higher workload exposures in training and/or matches without additional subjective strain could potentially facilitate factors such as progressive overload and skill execution under lesser perceptual strain, and may increase players’ capacity to perform work during competition should this be desired. Moreover, enhanced psychophysiological recovery could permit shorter turnaround times between high‐load sessions, potentially reduce fatigue/muscle damage‐related injury risk, support higher training quality (e.g., players could theoretically undertake a greater volume/intensity of tactical work in a session immediately preceding a match) whilst incurring a lower fatigue response (Caru et al., 2019; Daab et al., 2025). Nevertheless, practitioners must also consider that this attenuated fatigue/accelerated recovery may potentially come at the expense of blunted adaptive responses, potentially in a similar manner to other modalities which can result in accelerated recovery (e.g., cold water immersion, compression garments) (Brown et al., 2023). For instance, although the potentially blunted inflammatory responses associated with IPC may attenuate post‐exercise soreness and muscle damage, inflammatory signalling may also represent an important process underpinning physiological adaptation. Indeed, anti‐inflammatory medications have been documented to attenuate adaptive responses in young healthy participants (Lundberg & Howatson, 2018). Therefore, all considered, practitioners may carefully consider the context of potential IPC application, along with its acute and potential longitudinal effects.

Readers may also consider that this study attempts to address a critical limitation of much IPC research, the lack of adequate placebo control (Hurst et al., 2020; Sabino‐Carvalho et al., 2017). Consequently, it follows that many of the effects are likely smaller than others which did not attempt to adequately blind participants. Further, the nature of the study design, wherein the session involved an ecologically valid in‐season session, is likely to result in less muscle damage then other extensive protocols, rendering effects more challenging to detect. However, by using a real‐world in‐season session, this approach more closely reflects the likely practical application of IPC, thereby providing practitioners with contextually relevant insights. Further, by employing a credible sports supplement placebo rather than a low‐pressure cuff sham, we attempted to mitigate the blinding issues highlighted previously (Marocolo et al., 2025; Sabino‐Carvalho et al., 2017). Given that placebo effects in sport can yield performance gains of similar magnitude to IPC's average effect size (Brown et al., 2023; Marocolo et al., 2015), this design feature is essential for accurately estimating IPC's true physiological impact. Nevertheless, the present findings should be interpreted in light of important considerations. First, while the increased high‐intensity workload and recovery benefits are promising, whether these translate to other contexts, such as among female players or competitive match‐play performance outcomes, remains to be established. Secondly, the modest sample size may reduce statistical power and widen the uncertainty surrounding effect estimates, potentially as reflected in the generally anecdotal‐to‐moderate Bayesian evidence, although the study design helped mitigate statistical power reduction (Kowalski et al., 2025).

Importantly, it is noteworthy that individual responses varied, with some players demonstrating trends opposite to the group mean. Such variability is typical in team sport research and likely reflects the stochastic nature of intermittent team sport activity, which is influenced by contextual factors such as tactical involvement, opponent movements and transient physiological/psychological states and position. Regarding the latter, players occupied different roles, which may have influenced movement patterns during the small‐sided game component of the session. However, the counterbalanced crossover design helped account for this variability, considering that all drills, positional allocations and durations were standardised across conditions.

Accordingly, future work should consider larger samples and/or repeated observations to better account for this variability and improve effect estimate precision. In addition, work should (i) apply additional measures (e.g., isometric strength, blood markers, EMG) for greater mechanistic insight, (ii) examine different application timing and frequency of IPC to seek a potentially stronger effect, (iii) investigate whether repeated longitudinal IPC exposures produce cumulative effects with regard to blunted or enhanced physiological adaptations, and finally (iv) attempt to replicate the current observations, as well as determine effects in females.

### 4.1 Conclusion

The current findings, derived from randomized, double‐blind, placebo‐controlled crossover data in Gaelic football players, suggest that IPC may be used to enhance on‐field work capacity whilst simultaneously limiting acute perceptual strain and accelerating short‐term recovery. Therefore, team sport practitioners may consider implementing repeated IPC exposures (e.g., three 5‐min occlusions at 220 mmHg) to potentially facilitate (i) greater high‐intensity running outputs, (ii) lower acute fatigue responses and perceptual disturbances, and (iii) support subsequent recovery of physical function and perceived fatigue/soreness in the day(s) post‐exercise.

Practitioners should consider, however, that given the theoretical risk that routinely blunting inflammatory/fatigue signalling may attenuate longer‐term adaptations (Lundberg & Howatson, 2018), IPC perhaps should be periodized rather than used indiscriminately. For instance, the strategy could be prioritized for key training or competition macrocycles, when acute workload tolerance, readiness, or recovery for a short competitive turnaround are principal goals. Finally, this work demonstrates that remote, supervised delivery (e.g., convenient online compliance checks) appears feasible and minimally disruptive for players, eliminating the need for additional travel, time away from home or specialist facilities. Compared with commonly used recovery methods such as cold‐water immersion, IPC is also relatively comfortable and minimally disruptive, further supporting its feasibility as a practical option.

Given the inherently stochastic nature of team sport activity and subsequent response variability, these findings should be interpreted with appropriate caution. However, the collective directional consistency of observed effects, reflecting increases in workload alongside reductions in fatigue and enhanced recovery, render tentative confidence in the potential benefits of IPC applied in team sport.

## AUTHOR CONTRIBUTIONS

Lorcan Daly developed the study concept, led the writing of the manuscript, contributed to data collection, conducted the statistical analyses and produced the data visualisations. Shane Farrell was responsible for data collection, supported data/results organisation and management and recruited the participants. Alexander Gamble contributed to the study design, supported data organisation and provided critical input during manuscript revision. All authors were involved in interpreting the findings and reviewed and revised the manuscript. All authors have read and approved the final version of this manuscript and agree to be accountable for all aspects of the work in ensuring that questions related to the accuracy or integrity of any part of the work are appropriately investigated and resolved. All persons designated as authors qualify for authorship, and all those who qualify for authorship are listed.

## CONFLICT OF INTEREST

The authors declare no conflicts of interest.

## FUNDING INFORMATION

This work was not supported by any funding. All equipment used in this study, principally of note the IPC cuffs under examination, were purchased independently. No equipment, funding or support was provided by any commercial entity.

## Data Availability

The data underpinning this research are openly accessible at an open science framework repository link (https://tinyurl.com/3hncnn9a), thereby promoting transparency/reproducibility. This dataset is made available for practitioners and researchers who may wish to reanalyse or validate it for further investigation.
